# Thanks to Reviewers 2022

**DOI:** 10.5334/pb.1205

**Published:** 2023-02-02

**Authors:** Editorial Team

**Affiliations:** 1 Psychologica Belgica, BE

## Abstract

All manuscripts published in Psychologica Belgica have been assessed conscientiously and unselfishly by expert reviewers. The quality of our journal totally depends on their valuable and constructive criticisms to the authors. Both the editors and the authors highly appreciate the input and dedication of all our reviewers. Many thanks.

Mohammed Atari

El-Sayed Baz

Joël Billieux

Pavel Blagov

Guillermo Borragan Pedraz

Dries Bostyn

Lieven Brebels

Poppy Brown

Yohanes Budiarto

Emma Cernis

Fabienne Collette

Otto Condliffe

Manon Couvignou

Emiel Cracco

Deborah De Moortel

Hans De Witte

Mathieu Declerck

Bruno Delepierre

Kobe Desender

Aziz Essadek

Wim Fias

Medhi Gilson

Leen Haerens

Michel Hansenne

Marie Helweg-Larsen

Frank Laroi

François Léonard

Isabelle Milhabet

Sofie Morbée

Géraldine Petit

Pierre Philippot

Karolien Poels

Julia Schnepf

Linda Shanock

Or Shkoler

Bart Soenens

Samuel Suarez

Nette Vandenhouwe

Tim Vanhoomissen

Cato Waerloos

David Zuroff

